# Analysing the sensitivity of nestedness detection methods

**DOI:** 10.1007/s41109-017-0057-9

**Published:** 2017-10-23

**Authors:** Alexander Grimm, Claudio J. Tessone

**Affiliations:** 1 University Research Priority Program Social Networks, University of Zurich, Andreasstrasse 15, Zurich, Switzerland; 2 Department of Business Administration, University of Zurich, Andreasstrasse 15, Zurich, Switzerland

**Keywords:** Nestedness, Networks, Sensitivity, BINMATNEST, NODF

## Abstract

Many bipartite and unipartite real-world networks display a nested structure. Examples pervade different disciplines: biological ecosystems (e.g. mutualistic networks), economic networks (e.g. manufactures and contractors networks) to financial networks (e.g. bank lending networks), etc. A nested network has a topology such that a vertex’s neighbourhood contains the neighbourhood of vertices of lower degree; thus –upon vertex reordering– the adjacency matrix is *step-wise*. Despite its strict mathematical definition and the interest triggered by their common occurrence, it is not easy to measure the extent of nested graphs unequivocally. Among others, there exist three methods for detection and quantification of nestedness that are widely used: BINMATNEST, NODF, and fitness-complexity metric (FCM). However, these methods fail in assessing the existence of nestedness for graphs of low (NODF) and high (NODF, BINMATNEST) network density. Another common shortcoming of these approaches is the underlying assumption that all vertices belong to a nested component. However, many real-world networks have solely a sub-component (i.e. a subset of its vertices) that is nested. Thus, unveiling which vertices pertain to the nested component is an important research question, unaddressed by the methods available so far. In this contribution, we study in detail the algorithm *Nestedness detection based on Local Neighbourhood* (NESTLON). This algorithm resorts solely on local information and detects nestedness on a broad range of nested graphs independently of their nature and density. Further, we introduce a benchmark model that allows us to tune the degree of nestedness in a controlled manner and study the performance of different algorithms. Our results show that NESTLON outperforms both BINMATNEST and NODF.

## Introduction

Within a network, two vertices are nested with each other if the neighbourhood of the one with larger degree contains the neighbourhood of the one with lower degree (Mahadev and Peled 1995). We call *nested component* (Grimm and Tessone 2017) of a network the largest set of vertices that are nested (pairwise) above statistical significance. Following, a network is nested if the extent of the nested component is such that it embraces all vertices. This definition applies in both bipartite and unipartite networks^1^. In general, nested networks generalise trivial topologies such as stars or cliques and (for low densities) are largely hierarchical. In real-world networks that display this property, some edges violate the pairwise definition of nestedness given above; in this case, the lower the number of these violations, the larger the degree of nestedness of the network.

Importantly, nestedness is a common feature for many real-world networks in the most variegated realms. Already in mid-last-century, in the field of biogeography, it was recognised that species and biotas (Ulrich et al. 2009) form a bipartite network with this property. However, the most prominent example for nestedness is found in ecological networks. As it was discovered in the last decade - and intensively studied since - mutualistic networks show a pronounced degree of nestedness (Bascompte and Jordano 2013; Bascompte et al. 2003). A broad range of mutualistic networks show a nested structure: plant-pollinator (Ulrich et al. 2009) (i.e. ranging from marsh land to subarctic regions) in disparate regions (i.e. ranging from Australia to Greenland), host-parasite (Toju et al. 2014; Guimarães and Guimarães 2006; Worthen and Rohde 1996), seed-dispersal (Bascompte et al. 2003; Hernández et al. 2017), etc. In all these networks, connections exist only between species of one class to species of the other (e.g. a pollinator is connected to a plant, if it can pollinate it). Highly connected nodes are called generalists, and sparsely connected ones are called specialists. In nested networks, generalists connect to both specialists and generalists, while specialists only connect to generalists, i.e. they create a very specific core-periphery structure.

Beyond its natural field of application, nested network arrangements are a common occurrence in socio-economic networks: For example, the bipartite networks of manufacturer-contractor in the garment industry show this property for very extensive periods of observation (Uzzi 1996). The same was found for the network of products manufactured in a country (Tacchella et al. 2012), and the scientific domains in which countries are active (Cimini et al. 2014); in both examples, countries are connected to products (scientific domains) if they are produced (developed) there.

But also unipartite networks show the property of nestedness: For example, this was found for inter-country trade relations (import-export of arbitrary goods but also arms-trade network) (König et al. 2014), and loans in the inter-bank market (Soramäki et al. 2007). In the field of management, nested inter-firm networks were shown to facilitate knowledge growth in interconnected models, and empirical evidence of this network trait found in disparate industry sectors (Tomasello et al. 2016). Lately, auction and bilateral negotiation fish markets were shown to be nested (Hernández et al. 2017).

Four methods have gained particular attention for detecting and quantifying nestedness in the last decade: *Binary matrix nestedness temperature calculator* (BINMATNEST) (Rodríguez-Gironés and Santamaría 2006), based on the *Nestedness Temperature Calculator* (NTC) (Atmar and Patterson 1993), *Nestedness metric based on overlap and decreasing filling* (NODF) (Almeida-Neto et al. 2008), and *Fitness-Complexity Metric* (FCM) (Tacchella et al. 2012). Nonetheless, these methods detect nestedness only on specific density ranges (BINMATNEST, NTC and NODF fail in detecting nestedness for high density networks) or a specific class of graphs (FCM was developed only for bipartite ones).

All four methods assume that all vertices belong to a single nested component but, in general, this is not necessarily true. Such a nested component might include solely a subset of vertices while the others (although included into the network dataset) may not be part of it. Therefore, devising a method that identifies the vertices that belong to a nested component is an important methodological advance, which previous methods have not addressed.

The widely used BINMATNEST is based on NTC, which compares the focal adjacency matrix with a “perfect ordered” matrix. The less these two matrices deviate from each other, the more the graph is judged as nested. However, the matrix of “perfect order” is a normative concept characterised by a static (largely arbitrary) isocline (Atmar and Patterson 1993) (i.e. matrix is filled up to the secondary diagonal). Both methods judge graphs only as nested if they have this particular “perfect order”. Therefore, they fail in detecting graphs that have locally nested components. Further, this normative concept of nestedness relies only on global information (i.e. irrespective of local neighbourhoods in the nested components). For large datasets it is important to develop methods for detecting nestedness that rely solely on local information, because their performance scales better (Grimm and Tessone 2017).

In this contribution we perform a sensitivity analysis of NESTLON (*Nestedness detection based on Local Neighbourhood*) that reliably detects nestedness irrespective of network density, type (i.e. bipartite and unipartite networks), and adjacency matrix shape (Grimm and Tessone 2017).

The remainder of the paper is organised as follows. In the next section “The notion of nestedness” we provide an overview about nestedness in graphs and the current methods for detecting it. In “Algorithm” section we review the alternative method NESTLON for detecting nestedness. In “Results: sensitivity analysis” section we compare the reliability of detecting nestedness on benchmark graphs among commonly used algorithms and NESTLON. The final section concludes and discusses the main contributions of this Paper.

## The notion of nestedness

### Definition of nestedness

We first give an informal definition of nestedness and later a mathematical one. The neighbourhood of a vertex is the set of vertices that it is connected to. Then, an unipartite network is said to be nested if for each vertex, its neighbourhood contains the neighbourhoods of vertices which have lower degree (König and Tessone 2011). For bipartite networks, a related definition applies, with the caveat that the neighbours must be of the same class (Bascompte et al. 2003). In abstract terms, a nested graph may be thought as easy to identify: A graph is nested if, when its nodes are sorted by descending degree, the adjacency matrix is step-wise. This means that the adjacency matrix of a perfectly nested network can be divided into two well-differentiated regions: an upper-left with the existing edges and a lower-right region without any (cf. Fig. 2, top left).

For a proper mathematical characterisation of nestedness we briefly recapture the nomenclature for graphs. The adjacency matrix, *A*, characterises the topology of a graph object *G*. A non-zero entry in the adjacency matrix, *a*_*ij*_≠0, indicates an edge between the two vertices *i* and *j*. Each vertex has a degree, *k*_*i*_, which is the number of neighbours it is connected to. The total number of edges is *e* and the total number of vertices is *n*. *N* is the set of all vertices and *E* is the set of all edges. A unipartite graph can be decomposed by the concept of degree partition (Mahadev and Peled 1995):

#### **Definition 1**

Let *G*=(*N*,*E*) be a graph whose distinct positive degrees are *k*_(1)_<*k*_(2)_<…<*k*_(*r*)_ and let *k*_(0)_=0 (even if no vertex with degree 0 exists in *G*). Further, define $\mathcal {D}_{i} = \{ \nu \in N:k_{\nu } = k_{(i)} \}$ for *i*=0,…,*r*. Then the set-valued vector $\mathcal {D} = (\mathcal {D}_{0}, \mathcal {D}_{1}, \ldots, \mathcal {D}_{r})$ is called the degree partition of *G*.

With the concept of degree partition a nested unipartite graph can be expressed as follows (Mahadev and Peled 1995):

#### **Definition 2**

Consider a nested graph *G*=(*N*,*E*) and let $\mathcal {D} = (\mathcal {D}_{0}, \mathcal {D}_{1}, \ldots, \mathcal {D}_{m})$ be its degree partition. Then the vertices *N* can be partitioned in independent sets $\mathcal {D}_{i}$, *i*=1,…,⌊*m*/2⌋, and a dominating set $\bigcup _{i=\lfloor {m/2}\rfloor +1}^{m}\mathcal {D}_{i}$ in the graph $G^{\prime } = (N \setminus \mathcal {D}_{0}, E)$. Moreover, the neighbourhoods of the vertices are nested. In particular, for each vertex $\nu \in \mathcal {D}_{i}, i=1, \ldots,m$, we obtain the sets of vertices *N*_*ν*_ (i.e. the neighbourhood of vertex *ν*) as

1 $$ N_{\nu} = \left\{ \begin{array}{ll} \bigcup_{j=1}^{i} \mathcal{D}_{m+1-j} & \text{if \(i=1, \ldots, \lfloor{m/2}\rfloor\)};\\ \bigcup_{j=1}^{i} \mathcal{D}_{m+1-j}\setminus\{\nu\} & \text{if \(i=\lfloor{m/2}\rfloor+1, \ldots,m\)}. \end{array}\right.  $$

in which the maximum degree is given by *m*.

An adjacency matrix of an unipartite graph is step-wise if the following definition holds (Brualdi and Hoffmann 1985):

#### **Definition 3**

A step-wise matrix *A* is a symmetric, binary (*n*×*n*) matrix with elements *a*_*ij*_ satisfying the following condition: if *i*<*j* and *a*_*ij*_=1, then *a*_*hk*_=1 whenever *h*<*k*≤*j* and *h*≤*i*.

Concluding, we call a graph nested if its adjacency matrix, ordered by descending degree, is step-wise and if we can find a degree partition separating its set of vertices into an independent and a dominating set.

As a generalisation, bipartite graphs have two disjoint vertex sets *U* and *V*. In particular, each edge connects a vertex in the set *U* with one in set *V*, but not two vertices within the same set. A bipartite graph can analogously be decomposed into one degree partition for each of the two sets *U* and *V* as we have shown it for unipartite graphs. Without loss of generality we can separate a bipartite graph into independent sets and a dominating set within each of the two disjoint degree partitions *U* and *V* independently.

Nestedness can be now formally expressed as a local (pairwise) concept between two vertices.

#### **Definition 4**

Two vertices *i* and *j* are pairwise nested if the following condition applies

2 $$ k_{i} \le k_{j} \wedge a_{j\ell} = 1 \Rightarrow a_{i\ell} = 1, \,\,\, \forall \ell   $$

#### **Definition 5**

A network is *fully nested* if for any pair of vertices of the same type, they are pairwise nested.

In general, real-world networks will not be fully nested: either missing edges within or unexpected edges outside the expected structure will likely occur. By looking at the adjacency matrix the former appear as holes, whereas the latter appear as scattered dots. The number of these *violations* to the nested condition serve indeed as a straightforward quantification of the quality of the nested structure. In the following we will quantify the total number as well as the density of these violations.

#### **Definition 6**

The number of violations of pairwise nestedness between two vertices *i* and *j* is given by:

3 $$ \eta(i,j) := \Theta(k_{i} - k_{j}) \sum\limits_{\ell \leq N} a_{j\ell} \cdot (1 - a_{i\ell}),   $$

where *Θ*(·) is the Heaviside function.

Each term in the summation of Eq. 3 is equal to one for vertices *ℓ* connected to the lowest degree node, but not connected to the larger degree node.

#### **Proposition 1**

Given that *a*_*jl*_ equals one *k*_*j*_ times and (1−*a*_*il*_), (*n*−*k*_*j*_) times, the maximum number of violations of pairwise nestedness between two vertices *i* and *j* is given by:

4 $$ \eta_{max}(i,j) = \Theta(k_{i} - k_{j}) \min(k_{j}, n-k_{i})   $$

Now, counting the number of violations normalised by its maximum possible value, it is possible to derive a global measure for the density of violations in the entire network.

#### **Proposition 2**

The density of violations *γ*_*v*_ in a network is given by the normalised number of pairwise nestedness violations. The summation is restricted to vertex pairs for which *k*_*i*_≥*k*_*j*_ because the sums for the excluded cases are empty.

5 $$ \gamma_{v} = \frac{{\sum\nolimits}_{i,j\in N} \Theta(k_{i} - k_{j}) {\sum\nolimits}_{\ell \in N} a_{j\ell} \cdot (1 - a_{i\ell})} {{\sum\nolimits}_{i,j\in N} \Theta(k_{i} - k_{j}) \min(k_{j}, n - k_{i})}   $$

While the previous holds for undirected networks, it can be trivially extended to directed ones.

### Detection and quantification of nestedness

In this section we briefly discuss methods most commonly used for quantifying nestedness in graphs. A naive approach to quantify the degree of nestedness is to compare the actual observed network with a perfectly nested one. The more the focal graph deviates from the perfectly nested graph, the less nested it actually is. And indeed, many methods of nestedness detection pick up the concept of deviation with respect a nested null model.

For example, *Graph Edit Distance* (GED) (Sanfeliu and King-Sun 1983) is such a measure for similarity between two networks. It counts the number of atomic operations (i.e. link rewiring) that are necessary to transform one network into another. However, it is evident that GED does not scale well on network size because of the combinatorial growth in the possible sequence of operations. It is, thus, for the practitioner impractical to use such method on most situations of interest (Neuhaus et al. 2006).

Another method using a similarity measure is the NTC (Nestedness Temperature Calculator). The matrix temperature *T* is a measure of how uniform is the distribution of edges across the adjacency matrix and is computed in three steps (Rodríguez-Gironés and Santamaría 2006). First, a parametric isocline (representing perfect order) is created separating the regions in the adjacency matrix with and without edges. Second, the adjacency matrix is reordered by permuting rows and columns in a way that it maximises the packing of edges in the upper-left part of the adjacency matrix. Third, the distances of the holes remaining above and unexpected entries below the isocline that still deviate from the perfect order. Then, the matrix temperature is the sum of these distances. Unfortunately, the NTC is dependent on a normative concept of a global null model (Ulrich et al. 2009), an arbitrary isocline (Guimarães and Guimarães 2006) and has no unique solution (Rodríguez-Gironés and Santamaría 2006).

The two approaches mentioned above result impractical because the number of possible permutations becomes extremely large in typical networks, therefore it is impossible to compute them all. With the NTC, for example, we have to find the one configuration that leads us to the maximum packing among *n*!*m*! others (in absence of duplicated rows and columns), (Rodríguez-Gironés and Santamaría 2006)). NTC uses a heuristic approach to reach optimal packing but it does not attempt to solve this problem (Rodríguez-Gironés and Santamaría 2006)). Having these concepts in mind we will discuss in the following the three most commonly used measures for nestedness, which are BINMATNEST, NODF, and FCM. For a more comprehensive discussion please refer to Ulrich et al. (2009).

#### Binary matrix nestedness temperature calculator (BINMATNEST)

The method BINMATNEST is based on NTC and uses a genetic algorithm for reordering rows and columns so that the packing of the edges in the upper-left part of the adjacency matrix increases, aiming at minimising the matrix temperature. Before starting, BINMATNEST orders columns and rows in a descending order and removes empty and completely filled ones. On this matrix the genetic algorithm is encoded by two so-called *chromosomes*, one accounting for the rows and columns. These chromosomes indicate the position that a focal row (or column) of the original matrix should take in a proposed solution. The genetic algorithm generates *offsprings* based on the well-performing solution by cross-breeding the chromosomes. A more exhaustive explanation can be found in Rodríguez-Gironés and Santamaría (2006).

If all edges are in the upper left corner the temperature is minimum (*T*→0). If all edges are equally distributed in the matrix the temperature is maximum (*T*→100, an arbitrary value). The normalised temperature of the adjacency matrix is given by the following expression Flores et al. (2012):

6 $$ BINMATNEST = \frac{100 - T}{100}  $$

If *B* *I* *N* *M* *A* *T* *N* *E* *S* *T*=1 (0) the matrix temperature will be minimal *T*=0 (resp. maximal *T*=100). BINMATNEST is limited to connected graphs for which it computes the *p*-values of an ensemble of random graphs having the same size and density.

#### Nestedness metric based on overlap and decreasing filling (NODF)

NODF was developed for bipartite networks of ecological systems (Almeida-Neto et al. 2008) but it is applicable to unipartite networks, too. This method is independent of row and column order since it computes the paired nested degree for each pair of both columns and rows. However, in contrast to BINMATNEST this method does not reshuffle the matrix. The nestedness for the whole matrix is the sum of nestedness degrees of all paired rows and columns normalised by the number of all pairs. The NODF metric assigns a value $M_{ij}^{H}$ to each neighbouring pair of vertices (*i*,*j*):

7 $$ M_{ij}^{H} = \left\{ \begin{array}{lr} 0, & \text{if}\ k_{i} = k_{j}\\ \frac{o_{ij}}{min(k_{i}, k_{j})}, & \text{otherwise } \end{array} \right..  $$

The total number of common edges among the two vertices *i* and *j* is given by *o*_*ij*_. The procedure is carried out for columns ($M_{ij}^{P}$) and rows ($M_{ij}^{A}$) in an analogous procedure. Finally, the total nestedness for square matrices is then given by Saavedra et al. (2011) for all columns *P* and all rows *A*:

8 $$ \text{NODF} = \frac{{\sum\nolimits}^{P}_{i<j}M_{ij} + {\sum\nolimits}^{A}_{i<j}M_{ij}}{\frac{P(P-1)}{2} + \frac{A(A-1)}{2}}.  $$

For unipartite graphs the number of columns *P* and rows *A* is identical.

An advantage of NODF is its independence of matrix shape because it goes through both rows and columns (Saavedra et al. 2011) and of the exact matrix order. However, this method fails in quantifying nestedness for perfectly nested graphs according to their mathematical definition when network density is either low or high, because all terms involving vertices with the same degree cancel each other out.

#### Fitness-Complexity Metric (FCM)

FCM ranks vertices in an iterative and non-linear process (Tacchella et al. 2012). The iteration process couples a fitness term to a complexity term. Since FCM was solely developed for bipartite networks, we will not compare it to the above-mentioned methods in this contribution.

### Benchmark graphs

For comparing sensitivity and reliability among different nestedness detection methods we require a solid benchmark framework. A benchmark graph must allow the modification of important network characteristics in a controlled manner, which are network density, degree distribution, extent of nestedness, etc. In particular, we look for a synthetic backbone graph, which has a deterministic profile and keeps the degree distribution constant, and has stylised properties similar to those found in real-world systems.

A common way of creating nested networks is through *threshold graphs* (Chvátal and Hammer 1977; Mahadev and Peled 1995): for which, sequentially, new vertices are added; with probability *p* the vertex is isolated, and with the complementary probability 1−*p* it gets connected to all other existing vertices. All nested networks can be generated with finite probability by means of a threshold graph. However, for the purpose of this paper, threshold graphs are unsuited because the ensemble of graphs created is too diverse: Even for a given value of *p*, the degree distribution is stochastic and the level of fluctuations cannot be neglected. These fluctuations are stronger in the important case of very low density networks (low values of *p*), where the size of the dominating set is highly variable from realisation to realisation.

An alternative approach would be to provide a fixed degree sequence, which would make the threshold graph deterministic. However, it would imply the selection of a vector of values, determining the degree sequence.

To avoid these drawbacks, we resort on a generation process that generates deterministic degree distributions for a given parameter set and which is computationally efficient. In refs. König and Tessone (2011); König et al. (2014) it was shown that a network formation process where agents aim at maximising their centrality, naturally generates nested graphs with a single exogenous parameter *α* that influences the topology of the generated graphs fundamentally. This network formation process has two contrasting dynamics, edge creation and edge severance. First, the edge creating dynamics allows each vertex to create an edge to the most central vertex in its second-order neighbourhood (i.e. the neighbours of its own neighbours) with a probability *α*. Second, each vertex may severe the edge to the least central neighbour in its first-order neighbourhood with the complementary probability (1−*α*).

Regardless of the specific system for which this model was intended, the resulting networks are fully nested, with the parameter *α* controlling the network density. By changing *α* we can tune a nested graph between two limiting cases. On the one hand, we obtain a star topology for *α*→0 and, on the other hand, we obtain a fully-connected graph for *α*→1. A first-order phase transition exists at the critical value *α*=1/2 (König and Tessone 2011). For finite networks, by increasing *α* the matrix filling (i.e. the network density *γ*_*d*_) increases monotonically. Typically, non trivial nested topologies exist for |*α*−1/2|∝1/*n*. The benchmark graphs are nested by definition for every value of *α*∈[0,1]. The degree partition for the set of independent nodes is given by König et al. (2014)

9 $$ n_{k} = \frac{1-2\alpha}{1-\alpha}\left(\frac{\alpha}{1-\alpha} \right)^{k}  $$

In this paper we utilise this scheme for creating the benchmark networks as a starting point. After creating a nested component, this structure is *weakened* by random rewiring of edges. We use a randomisation process which keeps the degree sequence constant: First, a vertex is chosen at random (with equal probability). Then, an edge originated in this vertex is randomly chosen for removal and the focal vertex is connected to another one to which it was not already connected to. By doing so, we preserve the degree distribution with respect to in-degree. The total number of rewired edges *e*_*rew*_ is given by *e*_*rew*_=*ρ*_*rew*_·*n* in the sense that the higher is *ρ*_*rew*_, the more edges are randomly rewired. If this vertex is isolated or if it is connected to all vertices in the network, nothing happens. This process can be seen as an abstract representation of a model where the emergent network has some degree of nestedness (Bardoscia et al. 2013).

In absence of rewiring (i.e. *ρ*_*rew*_→0) the graph is almost perfectly nested and, thus, we expect that any quantification of nestedness should remain close to its maximum. Increasing the amount of rewired links such quantification should decrease monotonically. How fast such changes are detected is an indication of the sensitivity of the method. It is worth noticing that most real world networks in the literature involve what could be termed as *small* network sizes (i.e. few hundred nodes). While the results we present are for network of this order of magnitude, the results do not change qualitatively for larger ones.

The above-mentioned benchmark model is for unipartite graphs, which have an adjacency matrix with the identical number of rows and columns. However, with a trivial extension we can generalise it for bipartite graphs. Upon creating a benchmark graph of size *n*_*a*_ for a unipartite case, the number of columns is extended to *n*_*b*_ (*n*_*b*_>*n*_*a*_) and the same rewiring process as described above was performed. The asymmetric number of vertices for the two sets of vertices makes it possible to investigate bipartite benchmarks, whose adjacency matrices do not necessarily have the identical number of rows and columns.

## Algorithm

In this section we briefly review the algorithm NESTLON as a method for detecting nested components (Grimm and Tessone 2017). The simplicity of the algorithm is originated in the fact that it follows closely the mathematical definition of nestedness. In contrast to optimisation approaches (e.g. BINMATNEST), which strive for finding the extreme of a global measure (i.e. Network Temperature) by local assignment, NESTLON locally verifies the extent to which pairwise nestedness is violated for a given node with respect to all others. It goes iteratively from higher to lower degree vertices without any need for graph partitioning. Because there is no global optimisation concept behind NESTLON it depends only on local information, which makes this novel procedure computationally efficient, it has an excellent resolution compared to others, (Grimm and Tessone 2017). In the following we introduce the idea behind NESTLON followed by a more detailed discussion about the algorithm.

Let us suppose an arbitrary vertex (e.g. the one with the largest degree) is a candidate to belong to the nested component. NESTLON evaluates whether the neighbourhood of such a candidate vertex includes the neighbourhood of lower degree vertices in an iterative manner (i.e. the inclusion of the two neighbourhoods up to a confirmation ratio *θ*_*nest*_). This evaluation is softened, in the sense that a vertex is considered to belong to the nested component if it respects the local definition of nestedness to an acceptable degree (i.e. the ratio of confirmations has to exceed a parameter *θ*_*con*_). If a node passes its evaluation, its neighbours will become new candidates for the iterative evaluation. The iteration finishes when the candidate list becomes empty. Notice that all steps involve only local information, so the crawling process is fast.

The method iterates through the connected component of a graph starting with the highest degree vertex and, therefore, is applicable on both bipartite and unipartite graphs. The procedure is analogous for either in-degree or out-degree (for simplicity we refer merely to the term degree in the following). Without any loss of generality, we use the algorithm on a graph that is sorted by degree, which is also part of other procedures (e.g. BINMATNEST). The algorithm performs the following steps subsequently:


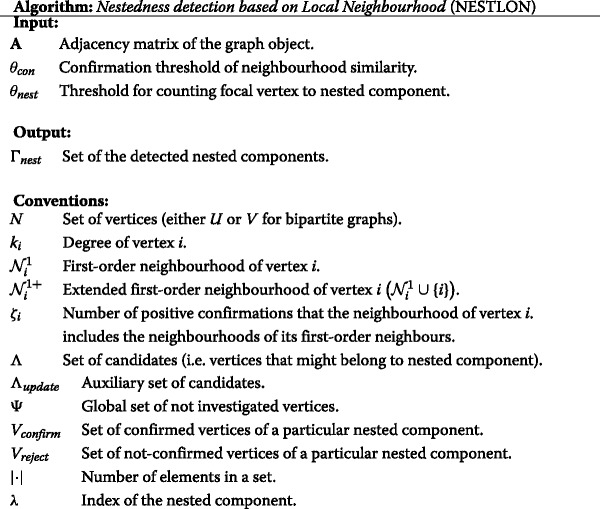



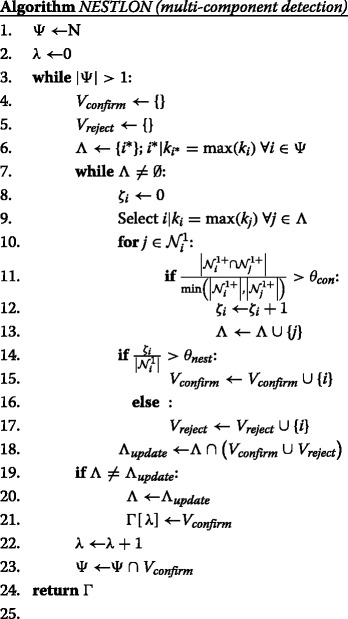


The outcome of the algorithm is a set of vertices, *Γ*, that belong to the *λ* nested components. A natural measure for the extent of the nested component is the ratio between number of vertices in the detected nested component and the maximum degree of the graph (as a measure for the largest hub originator of nestedness in the network).

10 $$ \mu_{NEST} = \frac{| \Gamma[\lambda^{\ast}] | }{ \max(k_{i})} \text{, with } i \in N \text{ and } \lambda^{\ast}|\,|\Gamma[\lambda^{*}]|=max_{\lambda\in[0,\dots,\lambda^{\text{max}}]}  $$

This method has several important features. First, it is independent on the adjacency matrix shape (i.e. ratio between number of rows and columns) and size (i.e. number of vertices). Second, in contrast to NODF it allows for identification of nestedness in rows and columns in an independent manner. Third, in contrast to NODF and BINMATNEST it can detect nested components irrespective of the graph density. Fourth, it can detect multiple nested components in a graph. We will now investigate the algorithm’s robustness and sensitivity in the next two sections.

### Results: NESTLON calibration

Before we can compare the algorithms among each other it is necessary to calibrate the two parameters of the NESTLON algorithm, *θ*_*con*_ and *θ*_*nest*_. The parameter *θ*_*con*_ is the confirmation threshold of neighbourhood similarity. The parameter *θ*_*nest*_ is the threshold for counting a focal vertex to the nested component. Both parameters are to be selected in a way that the size of the nested component equals the size of the largest connected components for networks where the nestedness property is respected for all pairs of nodes, but that – concurrently – allows a sizeable density of violations to nestedness.

#### Variation of *θ*_*con*_ and *θ*_*nest*_

**Fig. 1:**
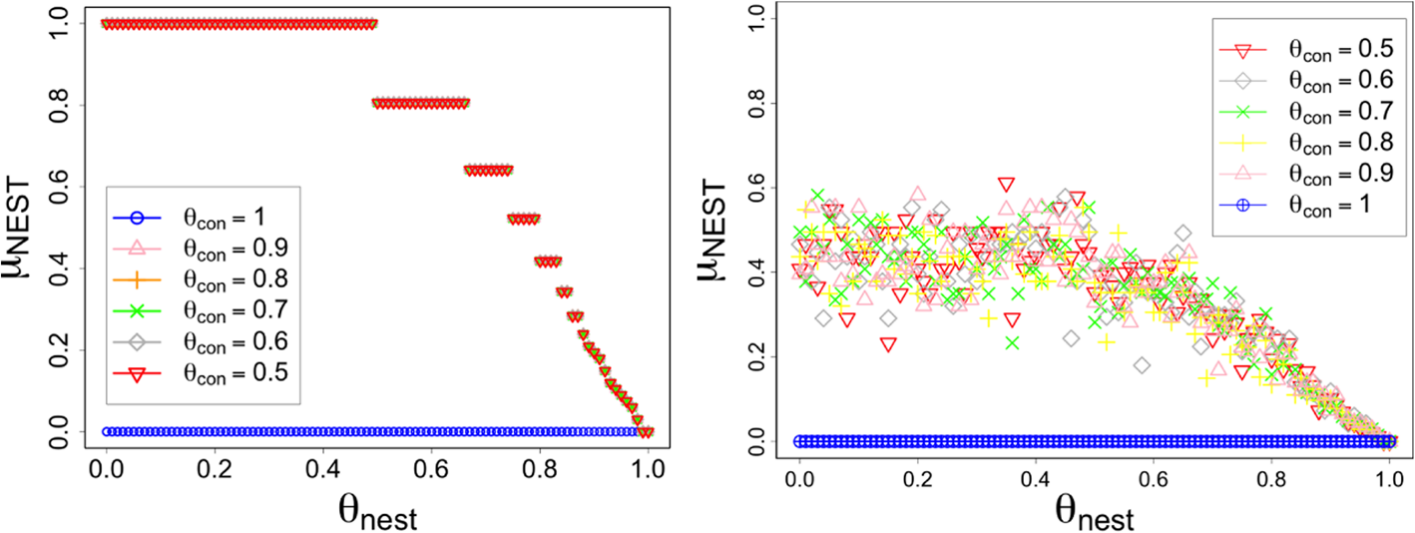
Nestedness for NESTLON under variation of both exogenous parameters *θ*_*con*_ and *θ*_*nest*_ on a perfectly nested unipartite benchmark graph (i.e. *α*=0.49). All vertices belong to a single nested component and, therefore, we choose a parameter set for which NESTLON detects all of them. In the left panel the thresholds are too rigid for *θ*_*con*_=1 and *θ*_*nest*_≥0.5. Thus, *θ*_*con*_<1 and *θ*_*nest*_<0.5 are reasonable detection thresholds for NESTLON. In the right panel we force NESTLON to start with a randomly selected vertex (in contrast to the highest degree) for the same benchmark graph. Although, component sizes differ independently from *θ*_*con*_<1 (about half of all vertices are detected on average due to random starting position), we infer the same calibration (*θ*_*con*_<1 and *θ*_*nest*_<0.5). We conclude that NESTLON works most reliable if we start with the highest degree vertex on a degree-ordered graph

In Fig. 1 we show the values of nestedness for the NESTLON algorithm under variation of both parameters *θ*_*con*_ and *θ*_*nest*_. The number of vertices that the algorithm counts as being nested does not differ for *θ*_*con*_<1 but decreases for *θ*_*nest*_≥0.5. Because we deal with a perfectly nested graph (i.e. benchmark graph with *α*=0.49, *ρ*_*rew*_=0) both parameters shall be set so that NESTLON measures full nestedness (i.e. $\mu _{NEST} \overset {!}{=} 1$). Thus, for these parameters we choose *θ*_*con*_<1 and *θ*_*nest*_<0.5 as reasonable for detecting nestedness for the next section. Notice that our benchmark allows also to sample arbitrary networks with a density of violation equivalent to any real dataset, to perform the same kind of analysis of the optimum parameters of NESTLON.

**Fig. 2:**
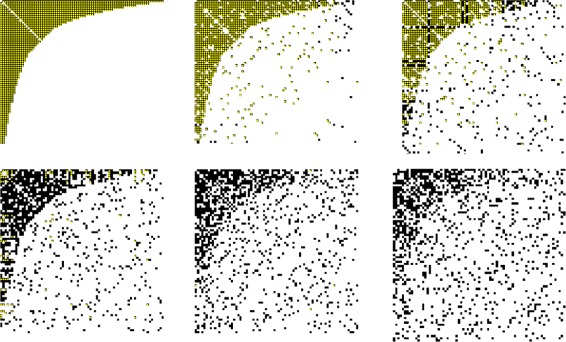
Adjacency matrices of the unipartite benchmark graphs with edge rewiring: *ρ*_*rew*_=0.0 (top left), *ρ*_*rew*_=1.0 (top center), *ρ*_*rew*_=2.0 (top right), *ρ*_*rew*_=3.0 (bottom left), *ρ*_*rew*_=5.0 (bottom center), *ρ*_*rew*_=7.0 (bottom right) for *α*=0.45. The vertices that are counted towards to the nested component by NESTLON are indicated by a yellow dot

#### Decreasing the level of nestedness

In Fig. 2 we show NESTLON’s ability in detecting the nested component on a benchmark graph with added random rewiring of edges. In absence of any rewiring (i.e. *ρ*_*rew*_=0) the algorithm includes all vertices as members of the nested component as desired. For increasing random rewiring (i.e. *ρ*_*rew*_>0) the algorithm counts fewer vertices as part of the nested component. This behaviour is expected because the graph looses its nested structure with increasing number of randomly rewired edges.

## Results: sensitivity analysis

A performing algorithm should detect the nested component independently of degree distribution, graph density, matrix shape, and matrix size. Such a solid algorithm should identify all vertices that fulfill the criterion of nested neighbourhoods (i.e. a higher degree vertex includes the neighbourhood of a lower degree vertex). In the following we compare the sensitivities of the three methods BINMATNEST, NODF, and NESTLON in detecting nestedness on unipartite and bipartite networks. For our analysis we use networks generated through the above mentioned benchmark graph.

### Unipartite networks

#### Increasing level of graph density

**Fig. 3:**
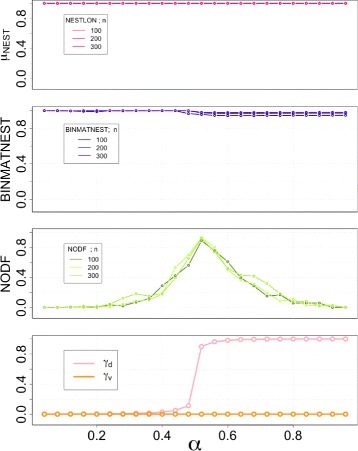
Robustness analysis for detecting the nested component among BINMATNEST, NODF and NESTLON on a benchmark graph. By definition all realisations of the benchmark graph are nested for all values of *α*. We perform the computation on a graph of size *n*=100,200,300. The graph density (i.e. *γ*_*d*_) increases with *α*, whereas the density of violations (i.e. *γ*_*v*_) remains around zero

In Fig. 3 we show the values of nestedness measured through the two standard methods BINMATNEST, NODF and the size of the nested component as detected by NESTLON for a unipartite graph constructed according to the benchmark above. The total network density is displayed in the lowermost panel, where the transition from very low density (highly hierarchical) to large density (homogeneous) networks is observed.

Although every graph constructed with the selected parameters is perfectly nested, BINMATNEST is not able to pack all edges into what it considers the upper left part of the adjacency matrix thereby creating a perfect packing. Therefore, for *α* larger than the phase transition (i.e. *α*>1/2) the quantification of nestedness becomes smaller than 1. This is because the genetic algorithm can not establish a better packing by reordering rows and columns in a fully connected network.

By performing the measurement of the degree of nestedness as detected by NODF, a more striking pattern is observed. While for very low and very large network densities the graph is perfectly nested, NODF gives a very low quantification of nestedness for the topology. Therefore, NODF fails in detecting nestedness for graphs with low (i.e. *α*<1/2) and high density (i.e. *α*>1/2). The reason behind this behaviour is that this method cancels out all rows and columns of same degree, therefore it induces a strong bias towards low nestedness for both low and high density graphs. The maximum level of nestedness is achieved for *α*≅1/2 where the model creates an upper-left diagonal matrix with density *γ*_*d*_=1/2. This nested network has the property that all degrees are different, i.e. *k*_*i*_=*i*−1 for *i*=1…*N*. The neighbourhood of a node with degree *k*_*i*_ is contained exactly in the neighbourhood of the node whose degree is *k*_*i*+1_, from which it always differs in a single vertex.

We use NESTLON to identify the nested component. The size of the nested component (see first panel of Fig. 3) indicates a completely nested network on a broad range of graph densities (i.e. *μ*_*NEST*_=1 for every value of *α*∈[0,1]).

#### Decreasing the level of nestedness

**Fig. 4:**
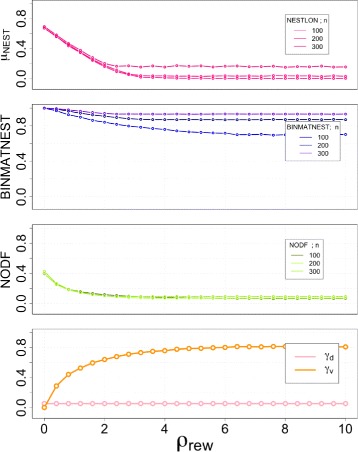
Robustness analysis for detecting the nested component among BINMATNEST, NODF and NESTLON on a benchmark graph with added noise. With increasing random rewiring *ρ*_*rew*_ the nested structure of the benchmark graph dissolves (i.e. increasing violation density *γ*_*v*_). We perform the computation on a graph of siz e *n*=100,200,300 and with *α*=0.45 (i.e. *γ*_*d*_≈0.029)

In Fig. 4 we analyse the level of nestedness as a function of the rewiring probability *ρ*_*rew*_. On the one hand, BINMATNEST is able to pack correctly the nested component, yielding a value close to 1 when *ρ*_*rew*_=0 (see second panel). On the other hand, NODF fails to measure the complete level of nestedness that exists in the network. This is particularly relevant as for very large *ρ*_*rew*_: in this limit the level of nestedness is negligible, but both measures still give a non-negligible quantification of the level of nestedness (cf. this Figure with Fig. 2). Further, both approaches have a very low sensitivity to the changes in nestedness in the system.

The violation density (Fig. 4) continuously increases for larger values of *ρ*_*rew*_ showing the continuous deterioration of the quality of the nested arrangement of edges in the network.

NESTLON has a different approach to nestedness detection: It selects the largest set of nodes that exhibit some level of nestedness. As it is apparent in the uppermost panel of Fig. 4, the size of the nested component continuously decreases as the number of rewired edges raises. This is in line with the intuition that in such a configuration, poorly connected nodes are simply connected by chance to the nested component, not belonging to it. The larger the disorder the more of these nodes exist. Indeed, for $\rho _{rew} \gtrsim 7$, the size of the nested component, as detected by NESTLON, vanishes.

### Bipartite networks

In this section, we perform an equivalent study for bipartite networks, where now it is also possible to analyse the role of the ratio in the number of rows and columns in the adjacency matrix. We change an adjacency matrix shape by simply varying the number of vertices belonging to either of the two sets in a bipartite graph, *n*_*a*_ (vertices belonging to the set *a* populating the rows) and *n*_*b*_ (vertices belonging to the set *b* populating the columns). In this contribution, without any loss of generality, we analyse *n*_*a*_>*n*_*b*_.

**Fig. 5:**
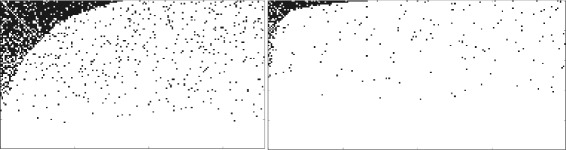
Examples of adjacency matrices of benchmark graph for *n*_*a*_/*n*_*b*_=1.5 (left panel) and *n*_*a*_/*n*_*b*_=3 (right panel) with addition one-dimensional random rewiring *ρ*_*rew*_=0.5. Due to the larger space for the right-hand plot the density is lower although the random rewiring has the same value

In both panels of Fig. 5 we show examples of two rectangular adjacency matrices of bipartite graphs under one-dimensional random rewiring with *n*_*a*_/*n*_*b*_=1.5 and *n*_*a*_/*n*_*b*_=3 respectively. In the following part, we calculate the level of nestedness of this benchmark graph and the size of the nested component as given by NESTLON while varying *α* and *ρ*_*rew*_ and changing adjacency matrix aspect ratio.

#### Increasing level of graph density

**Fig. 6:**
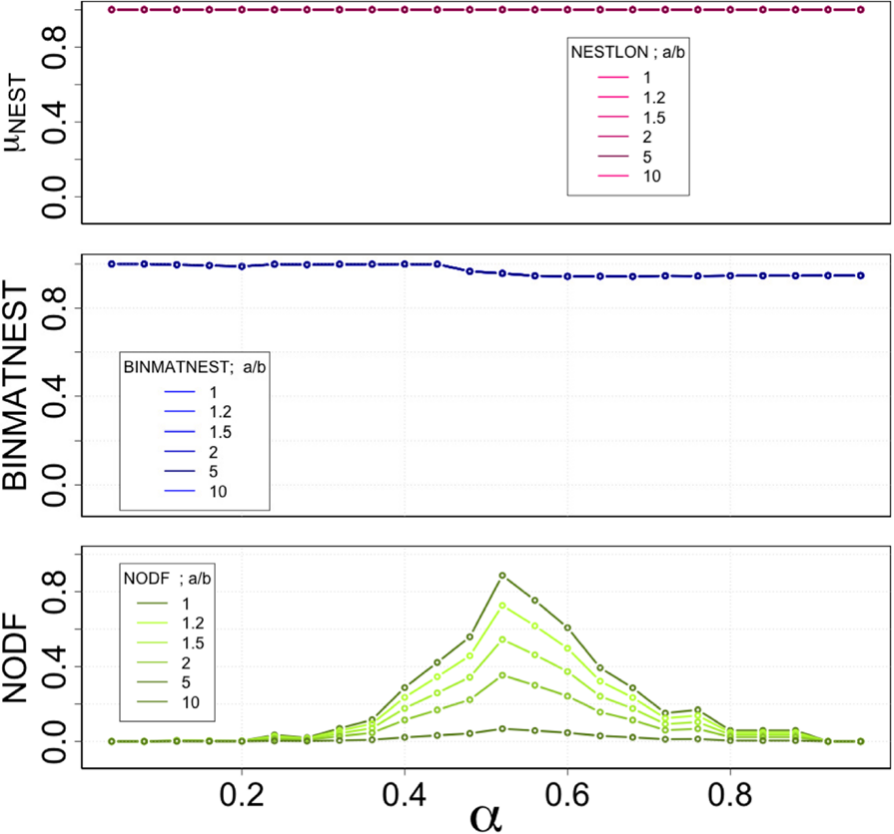
Sensitivity analysis among NESTLON, BINMATNEST, and NODF on a benchmark graph without added noise. The sensitivity in nestedness under the variation of the matrix shape (i.e. *n*_*a*_/*n*_*b*_) is much higher for the method NODF than for the two other methods BINMATNEST and NESTLON. We perform the computation on a bipartite graph of set size *n*_*b*_=100

The sensitivity analysis for different values of *α* (i.e. varying the network density) without edge rewiring is displayed in Fig. 6. The different curves represent changing values of the adjacency matrix aspect ratio *n*_*a*_/*n*_*b*_. BINMATNEST displays a similar behaviour as observed for unipartite networks: For *α*>1/2, the level of nestedness detected decreases marginally from full nestedness, which is detected for *α*<1/2. Interestingly, these results are unaffected by the actual aspect ratio. This is not true for NODF. The network density (which is also affected by the aspect ratio in our benchmark) changes completely the extent of nestedness measured by the widespread measure. Once again – as it occurred for unipartite networks – the size of the nested component detected by NESTLON is equal to the largest connected component in the network.

**Fig. 7:**
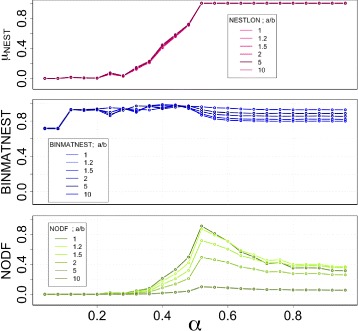
Sensitivity analysis among NESTLON, BINMATNEST, and NODF on a benchmark graph with added noise *ρ*_*rew*_=0.5. The sensitivity in nestedness under the variation of the matrix shape (i.e. *n*_*a*_/*n*_*b*_) is much higher for the method NODF than for the two other methods BINMATNEST and NESTLON. We perform the computation on a bipartite graph of set size *n*_*b*_=100

An additional analysis was performed in presence of a fixed proportion of rewired edges (i.e. *ρ*_*rew*_=0.5). The results are presented in Fig. 7. When the network density is very low (*α*≪1/2), any rewiring drives the node away form the nested component. The larger the network density, the less the rewiring process affects the nested topology. In any case, it is interesting to note that for largely connected networks, a lower level of nestedness exists for networks with a larger *n*_*a*_/*n*_*b*_ ratio.

#### Role of randomness in the networks

**Fig. 8:**
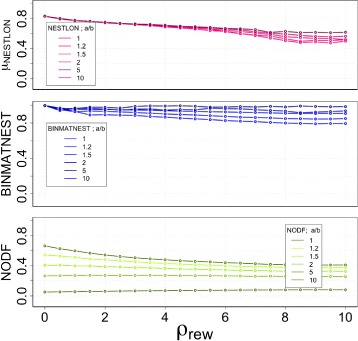
Sensitivity analysis among NESTLON, BINMATNEST, and NODF on a benchmark graph with an increasing fraction of rewired edges *ρ*_*rew*_. The values of nestedness are almost independent from the shape of the adjacency matrix for the method NESTLON. The variance in nestedness is larger for BINMANEST and NODF compared to NESTLON for differing matrix shapes, *n*_*a*_/*n*_*b*_={1,1.2,1.5,2,5,10}. The variance in nestedness against *ρ*_*rew*_ increases for the method BINMATNEST, whereas it decreases for the method NODF. We therefore judge NESTLON as least sensitive in detecting nestedness on bipartite graphs for a broad range of different matrix shapes. We perform the computation on a bipartite benchmark graph with *α*=0.49 and a set size of *n*_*b*_=100 and increasing *n*_*a*_

The sensitivity analysis for different values of *ρ*_*rew*_ is displayed in Fig. 8. By increasing the random rewiring *ρ*_*rew*_ the nested structure of the benchmark graph dissolves and, thus, a higher sensitivity in nestedness is necessary for a detection method.

Once again, the level of sensitivity of BINMATNEST and NODF is low: the former - regardless of the aspect ratio - detects high levels of nestedness. The latter detects vanishing values of nestedness for large aspect ratios, independently of the level of disorder that exists in the adjacency matrix. Importantly, for both quantification methods, given an aspect ratio, both are basically independent of the rewiring rate.

Importantly, the benchmark is built in such a way that the typical size of the nested component should be largely independent of the aspect ratio. NESTLON correctly exhibit this behaviour, as can be seen in the upper panel of Fig. 8. NESTLON is sensitive towards random rewiring in bipartite as well as unipartite graphs irrespective of the shape of the adjacency matrix.

## Conclusion

In this contribution we reviewed the novel method termed NESTLON for detecting nested components in graphs. As shown, widely-used algorithms such as BINMATNEST and NODF compute unreasonable low values of degrees of nestedness on benchmark graphs with either low density $\left (\text {i.e.}\ \gamma _{d} < \frac {1}{2}\right)$, NODF, or high density $\left (\text {i.e.}\ \gamma _{d} > \frac {1}{2}\right)$, NODF and BINMATNEST. The method NESTLON overcomes these limitations and is applicable on both bipartite and unipartite graphs irrespective of the shape of the adjacency matrix. NESTLON has a high sensitivity against *noisy* nested structures and a large robustness against different network densities. This shows that NESTLON is a solid method for detecting nestedness for a broad range of graphs. Importantly, NESTLON is purely based on the mathematical definition of nestedness and resorts solely on local information, which allows implementations of the algorithm to scale well on system size.

For the sensitivity analysis we created benchmark graphs with a network formation process. This benchmark allows us to tune the degree of nestedness and network density in a controlled manner. Our results show that techniques widely used to assess the level of nestedness of networks are largely insensitive to large structural changes in the network under analysis.

Widespread methods used to analyse the extent of nestedness in real-world networks assume that all elements of the dataset belong to what is defined here as the *nested component*. This contribution highlights that this *ansatz* cannot be given for granted, and that detecting the subset of nodes that actually belong to the nested component allows for a new characterisation of this ubiquitous network property.

## Endnote

^1^ in a slight abuse of notation - but for the sake of clarity - we call “unipartite”, those networks where all nodes are of the same class, to make clear contrast to bipartite networks.
